# Flow-controlled expiration reduces positive end-expiratory pressure requirement in dorsally recumbent, anesthetized horses

**DOI:** 10.3389/fvets.2023.1135452

**Published:** 2023-04-14

**Authors:** Jerrianne E. Brandly, Monica Midon, Hope F. Douglas, Klaus Hopster

**Affiliations:** Department of Clinical Studies, New Bolton Center, University of Pennsylvania School of Veterinary Medicine, Kennett Square, PA, United States

**Keywords:** equine, flow-controlled expiration, anesthesia, ventilation, respiratory, positive end-expiratory pressure, hypoxemia, atelectasis

## Abstract

**Introduction:**

Equine peri-anesthetic mortality is higher than that for other commonly anesthetized veterinary species. Unique equine pulmonary pathophysiologic aspects are believed to contribute to this mortality due to impairment of gas exchange and subsequent hypoxemia. No consistently reliable solution for the treatment of peri-anesthetic gas exchange impairment is available. Flow-controlled expiration (FLEX) is a ventilatory mode that linearizes gas flow throughout the expiratory phase, reducing the rate of lung emptying and alveolar collapse. FLEX has been shown to improve gas exchange and pulmonary mechanics in anesthetized horses. This study further evaluated FLEX ventilation in anesthetized horses positioned in dorsal recumbency, hypothesizing that after alveolar recruitment, horses ventilated using FLEX would require a lower positive end-expiratory pressure (PEEP) to prevent alveolar closure than horses conventionally ventilated.

**Methods:**

Twelve adult horses were used in this prospective, randomized study. Horses were assigned either to conventional volume-controlled ventilation (VCV) or to FLEX. Following induction of general anesthesia, horses were placed in dorsal recumbency mechanically ventilated for a total of approximately 6.5 hours. Thirty-minutes after starting ventilation with VCV or FLEX, a PEEP-titration alveolar recruitment maneuver was performed at the end of which the PEEP was reduced in decrements of 3 cmH_2_O until the alveolar closure pressure was determined. The PEEP was then increased to the previous level and maintained for additional three hours. During this time, the mean arterial blood pressure, pulmonary arterial pressure, central venous blood pressure, cardiac output (CO), dynamic respiratory system compliance and arterial blood gas values were measured.

**Results:**

The alveolar closure pressure was significantly lower (6.5 ± 1.2 vs 11.0 ± 1.5 cmH_2_O) and significantly less PEEP was required to prevent alveolar closure (9.5 ± 1.2 vs 14.0 ± 1.5 cmH_2_O) for horses ventilated using FLEX compared with VCV. The CO was significantly higher in the horses ventilated with FLEX (37.5 ± 4 vs 30 ± 6 l/min).

**Discussion:**

We concluded that FLEX ventilation was associated with a lower PEEP requirement due to a more homogenous distribution of ventilation in the lungs during expiration. This lower PEEP requirement led to more stable and improved cardiovascular conditions in horses ventilated with FLEX.

## 1. Introduction

General anesthesia in horses is associated with higher morbidity and mortality compared with other commonly anesthetized veterinary species (1, 2). In particular, horses undergoing prolonged procedures under general anesthesia are at an increased risk for the development of adverse events (1, 2). Associated anesthetic complications, such as hypoxemia, have been associated with an increased risk of postoperative complications such as surgical site infections (3) and poor recovery quality (4). Thus, the treatment of hypoxemia during general anesthesia is not only of critical importance for the immediate peri-anesthetic period but also for improvement of post-operative outcomes. Hypoxemia in horses under general anesthesia primarily develops secondary to compression atelectasis (5). The effects caused by the weight of the abdominal viscera on the diaphragm and thoracic cavity are more profound in horses in dorsal recumbency (6). In this position, the caudodorsal lung fields are at the highest risk for collapse (5, 6). However, these pulmonary areas remain preferentially perfused by relatively stiffer arteries that resist collapse (7), which results in significant ventilation/perfusion mismatching and right-to-left intrapulmonary shunting (8). The correlation between intraoperative atelectasis and the development of post-operative pulmonary complications, and their association with prolonged hospitalization, has been well established in humans (9, 10). While this correlation is less well defined in horses, pulmonary edema and pneumonia have been recognized as postoperative pulmonary complications in this species (11–13). Thus, ventilation strategies that improve pulmonary gas exchange and minimize atelectasis in horses under general anesthesia are needed to reduce intra-and postoperative complications.

Multiple mechanical ventilation strategies have been described to recruit collapsed lung tissue and improve oxygenation. Traditionally, these strategies have employed active lung inflation during the inspiratory phase and passive lung emptying during expiration. Studies have shown that high inspiratory pressures followed by sustained positive end-expiratory pressures (PEEP) can improve gas exchange in anesthetized horses (14–17). However, high inspiratory pressures can result in ventilator-induced lung injury (18, 19) and excessive PEEP can impair gastrointestinal perfusion (17) and cardiovascular function (20, 21). With the exception of PEEP-based techniques, optimization of ventilatory strategies to improve oxygenation and minimize lung-injury have focused on manipulation of the inspiratory phase (19, 22, 23). Recently, increased focus has been given to the modulation of the expiratory phase as an approach to lung-protective ventilation. One such modality, flow-controlled expiration (FLEX), linearizes the otherwise passive expiratory phase by reducing the initial high-expiratory flow and persisting expiratory gas flow throughout expiration (24, 25). In a porcine model of acute respiratory distress syndrome, FLEX reduced ventilation-induced lung damage, decreased the severity of pulmonary edema and focal inflammation, increased dynamic compliance, and improved ventilation (25). Furthermore, FLEX homogenized ventilation distribution by increasing ventilation in the dorsal/dependent lung regions in both lung-injured pigs (26) and lung-healthy human patients (27). Expiration is relatively rapid in patients with reduced lung compliance or with large body mass (18). The rapid expiratory flow that is generated results in increased shear stress that is greatest along the wall of small airways, resulting in their narrowing (28) and increasing the risk for collapse (25). This flow-related shear stress has also been postulated to contribute to mechanical signaling associated with ventilator-induced biotrauma (29). Additionally, the shear stress generated during cyclic alveolar recruitment-derecruitment perpetuates lung injury, further increasing the risk of lung collapse (30). A similar method of linearizing expiratory flow in humans resulted in improved regional ventilation in obese patients (31), and FLEX is likely of similar benefit in our larger veterinary species.

An initial investigation on the effects of FLEX on respiratory mechanics and gas exchange in horses yielded promising results (32). Hopster et al. (32) demonstrated that horses ventilated with FLEX had significantly higher PaO_2_ and C_dyn_ values, with no decrement in cardiovascular variables, compared to horses ventilated with conventional volume-controlled ventilation (VCV). Furthermore, switching from conventional VCV to FLEX resulted in a progressive increase in both arterial oxygenation and dynamic respiratory system compliance over time (32). However, this previous study did not incorporate an alveolar recruitment maneuver nor the application of PEEP in the experimental design. Earlier studies have shown a reduced PEEP requirement with FLEX ventilation in pigs (25) and in lung-healthy human patients (27).

Therefore, the aim of this study was to identify the effects of FLEX compared to traditional VCV on the lowest PEEP that still provided suitable improvements in dynamic respiratory system compliance and arterial oxygenation after alveolar recruitment in horses anesthetized in dorsal recumbency. We hypothesized that the FLEX modality would have improved respiratory system compliance and arterial oxygenation values compared to VCV. Also, FLEX ventilated horses would require a lower PEEP to prevent alveolar collapse, reflecting in a better hemodynamic function, compared to traditional VCV.

## 2. Materials and methods

A piston driven ventilator - Tafonius (Tafonius™, Hallowell EMC, Pittsfield, MA USA) Large Animal Anesthesia Workstation - was used in this study. As part of a previous study on FLEX ventilation in horses (32), the software of the machine was modified to perform FLEX ventilation by adding a linear release function. Image editing software (Phase Editor; Vetronics Services, Ltd) was used to program the ventilator to release the delivered tidal volume during the expiratory phase in a linear fashion. The inspired volume was measured, and the software calculated the flow needed to exhale at a constant rate such that it ends at the time of end-expiration.

The study was approved by the Institutional Animal Care and Use Committee of the University of Pennsylvania (protocol no. 806775-aaecgbc). A sample size calculation was performed *a priori* (power of 0.8 and an α-error of 0.05) showing that 6 horses per group would be necessary to detect clinically significant differences in cardiac output and level of PEEP, assuming a difference in CO of 6 l/min and difference in PEEP of 6 mmHg with a SD of 15% being clinically relevant (32, 33).

### 2.1. Animals

Twelve adult, healthy, university-owned horses were included in this prospective, randomized study. Horses were deemed healthy based on preanesthetic physical examination and had a mean ± SD body weight of (528 ± 63) kg and were between 4 and 19 years old. They were kept in stalls 72 h prior to anesthesia and fed hay. Food, but not water, was withheld 6 h before experimentation. Horses were randomly assigned to one of two treatment groups, VCV or FLEX, by use of a computerized random number generator.^1^

### 2.2. Instrumentation

Before induction of general anesthesia, the skin over both jugular veins was clipped and aseptically prepared for catheter placement. After infiltration of the skin with 2% lidocaine, a 12-gauge catheter (DayCath™, MILA International, Inc) was placed in the left jugular vein. Two 8 Fr catheter introducers (Exacta^®^ Percutaneous Sheath Introducer) were placed separately in the right jugular vein to facilitate placement of two balloon-tipped catheters. A Swan-Ganz standard thermodilution pulmonary artery catheter (Criticath™ 7 Fr/110 cm) was placed in the pulmonary artery and a second Swan-Ganz catheter into the right atrium for thermodilution cardiac output measurement. Correct placement was confirmed by visual inspection of the characteristic pressure waveforms.

### 2.3. Anesthesia

Horses were premedicated with 0.5 mg/kg xylazine (Rompun^®^ 100 mg/ml, Bayer Healthcare, LLC) IV and induced with 0.05 mg/kg midazolam (Midazolam HCL 50 mg/10 ml, West-Ward, INC) and 2.2 mg/kg ketamine (Zetamine™ Injection, MWI/VetOne) IV. Following induction of anesthesia, horses were orotracheally intubated with a cuffed Murphy endotracheal tube (Surgivet 24 mm ID, 32 mm OD x 90 cm) and subsequently positioned in dorsal recumbency on a padded surgical table. Anesthesia was maintained with isoflurane in oxygen (FiO_2_ > 95%), with an end-tidal inhalant concentration targeted at 1.4–1.6 Vol.% (34, 35). Intravenous crystalloid solution (Vetivex^®^ Veterinary pHyLyte™ Injection, Dechra Pharmaceuticals, PLC) was administered at a rate of 5 ml/kg/h and dobutamine (DOBUTamine HCl, Hospira, INC) was administered as a constant rate infusion at 1 mcg/kg/min as a minimum mean arterial blood pressure of 65 mmHg was needed to meet requirements for an unrelated surgical study the horses were enrolled in (unpublished data). A 20-gauge catheter (SURFLO^®^, Terumo Medical) was placed in either the left or right facial artery for invasive blood pressure monitoring and arterial blood sampling. The catheter was connected to a calibrated pressure transducer *via* rigid extension lines filled with heparinized saline and zeroed to atmospheric pressure at the level of the shoulder. As part of the concurrent surgical study, all horses were humanely euthanized for tissue harvesting and tissue sample collection *via* a dose of 4 mEq/kg potassium chloride administered IV at the end of anesthesia.

### 2.4. Experimental design

Following intubation and positioning in dorsal recumbency, the endotracheal tube was connected to the ventilator and all horses were immediately ventilated using a volume-controlled ventilation (VCV) mode with an inspiratory-to-expiratory (I:E) ratio of 1:2. The delivered tidal volume was set to 14 ml/kg (32, 36) throughout the experiment and respiratory rate was set at 6 breaths per minute then adjusted to maintain an end-tidal CO_2_ tension (PE’CO_2_) of 35–45 mmHg. The PEEP was set to 0 cmH_2_O. Horses assigned to group VCV were ventilated using conventional VCV with rapid, passive release of the airway pressure during expiration. The horses assigned to group FLEX were ventilated using the linear release function of the delivered tidal volume during expiration (32).

**Figure 1:**
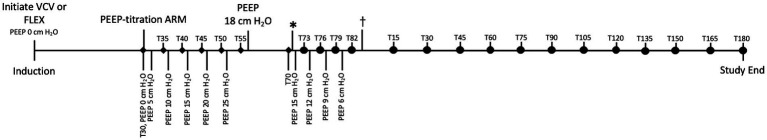
Timeline of study demonstrating sequence of events and timing of measurements. PEEP-titration alveolar recruitment maneuver (ARM) started 30 min after initiating ventilation (VCV or FLEX). During ARM, PEEP stepwise increased by 5 cmH_2_O every 5 min until 25 cmH_2_O reached. ♦: Mean arterial pressure (MAP), pulmonary arterial pressure (PAP), central venous pressure (CVP), heart rate (HR), and PaO_2_ measured and recorded just before PEEP adjusted. At completion of ARM, PEEP reduced to 18 cmH_2_O and maintained for 15 min. ^*^: PEEP stepwise reduced by 3 cmH_2_O every 3 min until alveolar closure pressure identified as indicated by a 20% decrease in PaO_2_ and C_dyn_. •: Indicates measurement and recording of MAP, PAP, CVP, HR, PaO_2_, and cardiac output (CO) just before each step of PEEP reduction. †: PEEP set at that which previously maintained PaO_2_ and C_dyn_. Measurements continued every 15 min until study completion. T30 through T82 corresponds to datapoints recorded on Table 1. T15 marks beginning of dataset recorded on Table 2.

Thirty minutes after initiating controlled ventilation (VCV or FLEX) a PEEP-titration alveolar recruitment maneuver was performed. While maintaining the set tidal volume, PEEP was stepwise increased by 5 cmH_2_O every 5 min until a peak PEEP of 25 cmH_2_O was reached, which was maintained for 5 min. Following the PEEP alveolar recruitment, the PEEP was reduced and set to 18 cmH_2_O and was maintained for 15 min. Then, the PEEP was stepwise reduced by 3 cmH_2_O every 3 min until the alveolar closure pressure was determined for both ventilation modes indicated primarily by a drop in PaO_2_, with or without a corresponding change in dynamic respiratory system compliance (C_dyn_) (Figure 1). A Pitot flowmeter (H-Lite, Morpheus Engineering, Netherlands) and a Cardiocap 5 monitor (CardiocapTM/5, Datex-Ohmeda, INC, United States) were used for respirometry, and the dynamic respiratory system compliance (Cdyn) was calculated by the monitor as C_dyn_ = VT_e_/(ΔP), where VT_e_ is the expiratory tidal volume and ΔP is the difference between the airway pressure at the two points of zero flow during the respiratory cycle (37). Peak inspiratory pressure (PIP) was measured by the integral multi-parameter monitor unit of the anesthesia workstation. Prior to each experiment, the Pitot flowmeter was calibrated with a three-liter calibration syringe (Ohio Cal-check 3-liter calibration syringe, Ohio Medical Products, USA). The 3 liters were injected at the same speed for each breath over 1–1.5 s, generating flows of approximately 150 to 180 l/min. The calibration factor, established from the mean values obtained over 10 consecutive breaths delivered using the calibration syringe, was 6.8 and was used to correct the Pitot flowmeter measured values. C_dyn_ values measured by the monitor were corrected by the calibration factor.

During the alveolar recruitment, the mean arterial pressure (MAP), pulmonary arterial pressure (PAP), central venous blood pressure (CVP), heart rate (HR), and PaO_2_ were measured and recorded before the PEEP was changed (Figure 1). After PEEP titration, the MAP, PAP, CVP, HR as well as cardiac output (CO) were recorded every 15 min. Measurement of CO by thermodilution was performed by manual injection of iced saline solution (1 ml/15 kg) through the catheter into the right atrium at end expiration. The temperature of the injectate was measured *via* an in-line temperature probe, and the temperature change in the pulmonary artery was analyzed to calculate the CO. Five injections were performed, and the average of the closest three CO values was used. Arterial blood was sampled before every CO measurement and was analyzed immediately with a blood gas analyzer (OPTI CCA-TS2 Blood Gas Analyzer, OptiMedical) to measure PaO_2_ and PaCO_2_.

The alveolar closing pressure was determined based on a drop of PaO_2_ of more than 20% (17, 38, 39). Once the alveolar closing pressure was identified, PEEP was titrated back to the previous level that maintained PaO_2_ and C_dyn_ for the remainder of anesthesia. Measurements were continued and data was recorded every 15 min.

### 2.5. Statistical analysis

Data was analyzed using the statistical software SAS 9.3 (SAS Institute Inc., NC, United States) and GraphPad Prism Version 7 (GraphPad Software, Inc. USA). Visual assessment of qq-plots and the Shapiro–Wilk test was used to confirm normal distribution of model residuals of dependent variables, and they are presented as mean ± SD for normally distributed data. Variables were compared to baseline (after alveolar recruitment) and between groups (at the same time points) using a two-factorial variance analysis for repeated measurements and Bonferroni correction for multiple comparisons and mixed-effects linear regressions. The level of significance was set to 5% (*p* < 0.05).

## 3. Results

Prior to the PEEP-titration alveolar recruitment maneuver, PIP ranged from 25 to 30 cmH_2_O. During the ARM at maximum PEEP, PIP ranged from 45 to 52 cmH_2_O.

**Figure 2:**
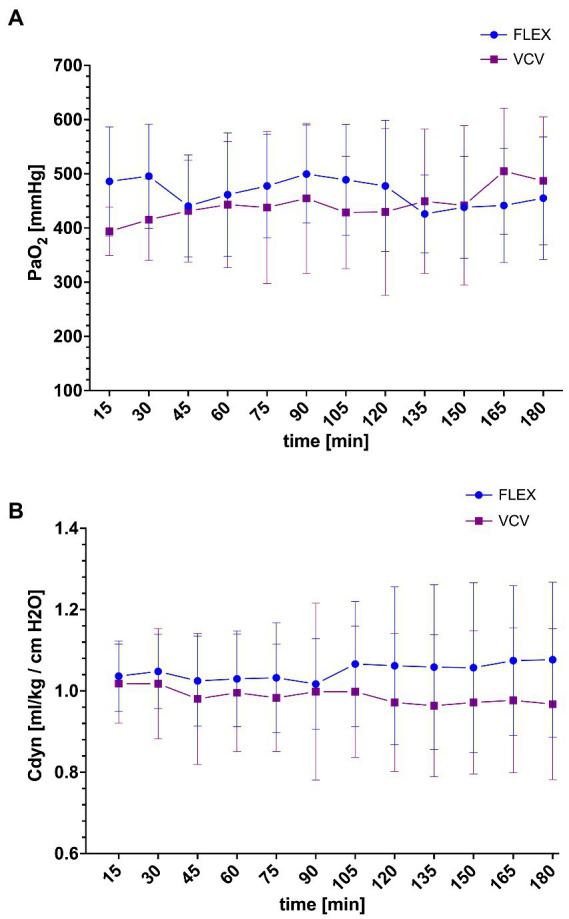
Mean and SD of the arterial partial pressure of oxygen (PaO_2_) **(A)** and weight-corrected dynamic compliance (C_dyn_) **(B)** in horses ventilated either with conventional volume-controlled ventilation (VCV, purple square) or with flow-limited exhalation ventilation (FLEX, blue circle) after PEEP-titration alveolar recruitment. T15 corresponds to T15 of Figure 1 and Table 2.

Following the equilibration period and prior to the PEEP-titration alveolar recruitment maneuver, the mean PaO_2_ and C_dyn_ of the horses were 137 ± 43 mmHg (range 118–184 mmHg) and 248 ± 98 ml/cmH_2_O (range 199–319 ml/cmH_2_O), respectively. After the alveolar recruitment, the PaO_2_ and C_dyn_ improved, ranging between 323–566 mmHg and 343–627 ml/cmH_2_O (Figure 2). After the decremental PEEP titration maneuver, the PaO_2_ and C_dyn_ remained stable and were not significantly different between groups (Figure 2).

The alveolar closure pressure was significantly lower for horses ventilated with FLEX (6.5 ± 1.2 cmH_2_O, 5 horses at a PEEP of 6 cmH_2_O and 1 horse at a PEEP of 9 cmH_2_O) compared to the VCV group (11.0 ± 1.5 cmH_2_O, *p* < 0.001, 2 horses at a PEEP of 9 cmH_2_O and 4 horses at a PEEP of 12 cmH_2_O). Therefore, horses ventilated with FLEX needed significantly less PEEP to maintain C_dyn_ and PaO_2_ compared to the VCV group (9.5 ± 1.2 vs. 14.0 ± 1.5 cmH_2_O).

The cardiovascular variables MAP, PAP, CVP and HR during the alveolar recruitment are shown in Table 1. There was no difference between groups nor change over time for any of the variables. The CO was significantly higher in the horses ventilated with FLEX at each time point compared to VCV (Table 2). The rates of dobutamine and fluids were the same for all horses over the course of the study period, with the total amounts administered not different between groups.

**Table 1:** Mean and standard deviation (SD) of mean arterial blood pressure (MAP), pulmonary artery pressure (PAP), central venous pressure (CVP) and heart rate (HR) of six horses ventilated either with FLEX or conventional volume controlled ventilation (VCV) during PEEP-titration alveolar recruitment; time point 0 min is connection to anesthesia machine.

Time	PEEP	MAP (mmHg)				PAP (mmHg)				CVP (mmHg)				HR (beats/min)				FLEX		VCV		FLEX		VCV		FLEX		VCV		FLEX		VCV		Mean	SD	Mean	SD	Mean	SD	Mean	SD	Mean	SD	Mean	SD	Mean	SD	Mean	SD
30	0	73	11	75	10	17	5	18	4	6	4	5	5	39	5	41	4
35	5	73	11	67	8	17	6	20	3	6	5	6	5	39	3	40	3
40	10	72	10	70	9	17	3	20	4	6	4	6	4	39	3	40	2
45	15	69	10	71	10	17	4	19	5	6	3	6	5	39	2	40	2
50	20	65	9	71	9	17	4	19	5	6	4	7	3	39	2	40	2
55	25	69	8	72	10	15	6	18	3	6	3	6	4	40	2	40	2
70	18	72	9	70	10	16	4	18	3	6	3	6	5	41	3	40	2
73	15	67	11	69	10	18	4	21	7	7	5	6	5	41	3	41	3
76	12	67	11	67	9	17	5	20	3	6	4	6	3	41	4	40	1
79	9	69	10	69^b^		15	3	23^b^		6	3	8^b^		42	4	44^b^	
82	6	71^a^	7			16^a^	4			6^a^	5			42^a^	4		

a 5 out of 6 horses in group FLEX.

b 2 out of 6 horses in group VCV.

* indicating statistically significant difference (*p* < 0.05) between groups FLEX and VCV at the same time point.

**Table 2:** Mean and standard deviation (SD) of mean arterial blood pressure (MAP), pulmonary artery pressure (PAP), central venous pressure (CVP), heart rate (HR) and cardiac output (CO) of six horses ventilated either with FLEX or conventional volume controlled ventilation (VCV) for 180 min after PEEP-titration alveolar recruitment; time point 0 min is set at after PEEP titration.

Time	MAP (mmHg)				PAP (mmHg)				CVP (mmHg)				HR (beats/min)				CO (l/min)					FLEX		VCV		FLEX		VCV		FLEX		VCV		FLEX		VCV		FLEX		VCV		*p*	Mean	SD	Mean	SD	Mean	SD	Mean	SD	Mean	SD	Mean	SD	Mean	SD	Mean	SD	Mean	SD	Mean	SD
15	73	11	70	10	17	5	18	4	6	4	5	5	39	5	41	4	37^*^	3	30	6	0.03
30	73	11	69	8	17	6	20	3	6	5	6	5	39	3	40	3	38^*^	4	29	6	0.02
45	72	10	70	9	17	3	20	4	6	4	6	4	39	3	40	2	38^*^	3	28	5	<0.01
60	69	10	71	10	17	4	19	5	6	3	6	5	39	2	40	2	37^*^	4	28	4	0.01
75	65	9	71	9	17	4	19	5	6	4	7	3	39	2	40	2	39^*^	5	29	6	0.01
90	69	8	72	10	15	6	18	3	6	3	6	4	40	2	40	2	37^*^	2	29	3	0.02
105	72	9	70	10	16	4	18	3	6	3	6	5	41	3	40	2	37^*^	3	27	5	0.03
120	67	11	69	10	18	4	21	7	7	5	6	5	41	3	41	3	37^*^	3	30	4	0.04
135	67	11	67	9	17	5	20	3	6	4	6	3	41	4	40	1	38^*^	5	29	5	0.03
150	69	10	69	9	15	3	19	5	6	3	6	5	42	4	40	3	37^*^	4	28	6	0.02
165	71	10	68	9	16	4	18	2	6	5	6	4	42	4	40	2	37^*^	5	28	4	<0.01
180	69	11	66	10	17	3	20	5	6	6	6	5	41	4	41	2	38^*^	4	29	4	0.01

* indicating statistically significant difference (*p* < 0.05) between groups FLEX and VCV at the same time point.

## 4. Discussion

This study was designed to build on previous research evaluating the effects of a flow-controlled expiration mode in anesthetized horses (32) by determining if horses ventilated with FLEX in dorsal recumbency would require less PEEP to maintain optimal respiratory mechanics and oxygenation. Indeed, horses ventilated with FLEX had a lower alveolar closure pressure than horses ventilated with conventional VCV and required less PEEP to maintain PaO_2_ and C_dyn_. Our findings indicated that less atelectasis occurred at lower airway pressures with FLEX ventilation, likely due to improved homogenization of ventilation and recruitment of dependent lung areas (26, 27, 31).

Mechanisms by which FLEX homogenizes ventilation and results in a better recruitment state continue to be investigated. Borgmann et al. (26) showed *via* electrical impedance tomography a redistribution of tidal ventilation from non-dependent to dependent lung regions in both healthy-lungs and lung-injured pigs ventilated using FLEX. It was further noted that the observed end-expiratory decruitment was less pronounced with FLEX, explained by the longer maintenance of alveolar pressure above closing pressure during expiration and shorter period of end-expiratory zero flow during which alveoli collapse (26). Ventilation strategies that manipulate the I:E ratio to shorten the expiratory phase and allow less time for alveolar collapse have been described (40). However, the shortened expiratory time increases the risk of auto-PEEP due to incomplete expiration (41). In contrast to these strategies, FLEX shortens the period of zero flow without changing the total duration of the expiratory phase.

The progression of passive expiration is largely dictated by the mechanical properties of the fast and slow compartments of the respiratory system (42). In a physical model of an inhomogeneous respiratory system, FLEX improved the pressure balance among compartments that would, in turn, reduce high local flow rates and shear forces within the lung tissue (43). The better-maintained airway expansion during expiration and more homogenized emptying of fast and slow compartments could explain the reduced PEEP requirement with FLEX, both in the present study and in previous reports (25, 27).

Horses ventilated with FLEX had significantly higher CO values after the PEEP alveolar recruitment. This is likely due to the lower PEEP requirement in this group of ventilated animals. It has been well established that the increased intrathoracic pressures associated with high positive inspiratory pressures and PEEP results in a reduction in cardiac output (14, 17, 20–22). This reduced CO is attributed to a decrease in right ventricular filling pressure, which results in a diminished stroke volume during increasing intrathoracic pressure (44). The lower peak and plateau airway pressures observed with FLEX (32) and significantly reduced PEEP requirement during FLEX ventilation, therefore, can explain the improved CO observed in these horses.

A limitation of this study is its randomized design. A cross-over design would have provided increased statistical power by allowing intra-individual comparison. However, this was not possible due to the terminal nature of the concurrent surgical experiment. While the small number of enrolled horses is an additional potential limitation of the study, a statistical *a priori* power analysis was performed, and the necessary number of horses were enrolled to ensure adequate power of the results which identified significant differences in the outcomes of interest. All horses were systemically healthy and normovolemic. Particularly in cardiovascularly compromised animals, the implementation of PEEP during ventilation can result in reduced CO and altered peripheral perfusion (45). Therefore, it is possible that in unstable critical equine cases even low levels of PEEP would not be tolerated. Additionally, although FLEX is associated with lower peak and plateau airway pressures, the longer maintenance of positive airway pressures during the respiratory cycle, reflected by higher mean airway pressures (26, 32), could negatively affect cardiovascular function. Although studies have demonstrated no significant detriment to cardiovascular variables in healthy patients (27, 32), this could be a potential limitation of FLEX ventilation and further studies are necessary to assess the safety of FLEX in cardiovascularly compromised patients. Similarly, none of the horses exhibited evidence of respiratory disease. While previous studies have investigated the effects of FLEX in lung injured patients in other species, additional studies investigating the safety and efficacy of FLEX in horses with respiratory pathology are warranted before its application in the clinical setting.

All horses received dobutamine during the entire experiment, which also further improved the blood pressure and CO (46). To better assess the effects of the ventilation modes on the cardiovascular system it would have been beneficial to investigate these horses without any cardiovascular support. However, due to the nature of the concurrent surgical investigation, the mean arterial blood pressure needed to be greater 65 mmHg and the CO greater 20 l/min in these horses to avoid severe compromise of the peripheral perfusion (17). However, the dobutamine infusion rate was kept constant and was used in all horses. Therefore, comparison between groups and assessment of trends is still possible.

A second alveolar recruitment maneuver was not performed after the initial PEEP titration maneuver to reverse the effects of the alveolar collapse that occurred. The lack of a second recruitment maneuver is anticipated to have minimal effect on the primary outcome of interest, which was the alveolar closure pressure for each of the ventilation modes. Adjusting PEEP back to the level that previously maintained PaO_2_ and C_dyn_ aimed to evaluate the ability of each of the ventilation modes to maintain the PaO_2_ and C_dyn_ as they were after the PEEP titration, while comparing hemodynamic variables during this maintenance phase. However, the lack of a second alveolar recruitment maneuver likely affected the magnitude of observed PaO_2_ and C_dyn_ values during the maintenance phase of anesthesia. Further, it has been shown that alveoli in peri-atelectatic regions experience increased mechanical stress (47) that results in increased alveolar disruption and inflammation in these areas. In mice, evidence of neutrophilic inflammation was already present in peri-atelectatic regions after 180 min of mechanical ventilation (48). While the time course of these changes has not been specifically evaluated in horses to the authors’ knowledge, Nyman et al. (5) had described hyperinflation and alveolar wall disruption within macroscopically normal lung areas after 2 h of mechanical ventilation a pony. Therefore, the atelectasis that started to occur at the end of the initial PEEP titration potentially contributed to further alveolar collapse. Although it was anticipated that any significant difference in the ventilation modes with regards to maintenance of pulmonary function still would have been detected considering neither group underwent a second alveolar recruitment maneuver, more subtle differences between the two ventilation modes in preventing further derangements in pulmonary function may have been better detected had a second alveolar recruitment been performed to leave the lungs more open and susceptible to atelectasis.

The literature regarding optimization of PEEP-titration alveolar recruitment maneuvers to reverse atelectasis and strategies for assessing alveolar closure is extensive and varied (49) with many utilizing changes in arterial oxygenation and respiratory system compliance. Suarez-Sipmann et al. (39) showed that during a decremental PEEP-titration maneuver the reduction in respiratory system dynamic compliance after reaching a maximum was related to the onset of end-expiratory lung collapse on computed tomography. In this study, the level of PEEP at which C_dyn_ first decreased corresponded to a 17% decrease from maximal PaO_2_. Continued reductions in PEEP resulted in continued reductions in arterial oxygenation that paralleled reductions in compliance (39). In human patients with early acute respiratory distress syndrome that underwent a maximum-recruitment followed by PEEP-titration maneuver, there was a high correlation between changes in PaO_2_ and the percent mass of collapsed lung on CT (50). Notably, however, these studies used PaO_2_/FiO_2_ (39) and PaO_2_ + PaCO_2_ (50) to assess the correlation between oxygenation and lung collapse, while here PaO_2_ alone was used. Because all horses received an FiO_2_ of 1.0, it is anticipated that the decrement in PaO_2_ alone rather than PaO_2_/FiO_2_ is suitable to make comparisons between ventilation modes. Results of these studies cannot be directly extrapolated to horses, however, as the correlation between PaO_2_ and alveolar collapse has been shown to be species dependent (51) and is less well described in horses.

The observed decrease in PaO_2_ and C_dyn_ during the decremental PEEP-titration in the present study occurred not always at the same level of PEEP. This is in agreement with previous studies. In anesthetized ponies that underwent an alveolar recruitment and PEEP titration, the PaO_2_ decreased earlier at higher PEEP levels than respiratory system compliance (14). Ambrisko et al. (38) similarly noted an earlier decrease in PaO_2_ than respiratory system compliance in anesthetized horses during the ramp-down phase following an alveolar recruitment. When the compliances of the non-dependent and dependent areas of the lung were evaluated separately on EIT, however, PaO_2_ significantly correlated to and changed with the compliance of the dependent lung (38). Only total respiratory system compliance was measured in the present study. Considering collapse of dependent lung areas in anesthetized horses is the major cause of shunt formation and decreased PaO_2_ (8), and the beneficial effects of FLEX on arterial oxygenation and respiratory system compliance (25, 32) are postulated to be due to the reduced end expiratory alveolar collapse and a more homogenized distribution of ventilation to dependent lung areas (26, 27, 31), PaO_2_ may also better correlate to the alveolar closure pressure of each of the ventilation modes in the present study.

The duration that each PEEP step was maintained may have influenced the results of this study. Studies using changes in PEEP every 2–3 min are described (52) and have been used to determine optimal PEEP in human patients with ARDS (53). However, these studies were not performed on horses. Although the authors noted that C_dyn_ stabilized within 1–2 min of each PEEP change in these horses, maintaining each PEEP step for longer may have identified a different alveolar closure pressure. Similarly, the earlier decrease in PaO_2_ at higher PEEP levels than C_dyn_ noted in the present study may also be due to the short duration that each PEEP step was maintained. While Ambrisko et al. (38) also noted an earlier decrease in PaO_2_ while maintaining each PEEP step for 10 min, there is the potential that C_dyn_ may have decreased at similar levels of PEEP had each PEEP step been maintained for longer in these horses.

The methodology for calibration of the pitot flowmeter is an additional limitation. The 3-liter syringe used for calibration of the pitot flowmeter likely did not generate ranges of all volumes and flows utilized for the size of horses in this study. However, flows similar to the inspiratory flow used in these horses were achieved, and although we cannot rule out that the calibration method used did not accurately reflect the study conditions, it was kept similar between experiments allowing comparison between groups. Additionally, airway pressures measured by the Cardiocap 5 monitor were not verified by a reference method. The Tafonius workstation measured airway pressures independently and although had showed good agreement in these horses and in a previous study (54), this was not specifically analyzed as part of the present study. The spirometry module of the monitor had been serviced 2 weeks prior to the start of the experiments, however, supporting accuracy of the pressure measurements. Regardless, potential inaccuracies in airway pressure and volume measurements obtained by the pitot flowmeter and monitor resulting from the calibration method and lack of verification with a reference method may have affected the C_dyn_ values, which were calculated as Vt_e_/(ΔP). Therefore, the C_dyn_ values obtained in this study may not directly reflect results obtained by other methodologies. With this in mind the authors elected to use the H-lite derived values for consistency to allow comparison between animals, and former published and unpublished data. As demonstrated previously, flow measurements by the H-lite demonstrate nonlinear behavior at higher flows (37) that could result in more significant variation in the error of volume and flow measurements with a large variation in body sizes. Due to the relatively smaller variation in the size of horses enrolled in this study, error in the flow and volume measurements associated with the calibration process represents a systematic error, and because the methodology used was kept consistent comparisons between treatments can be made.

Lastly, the results of this study may have differed if a double circuit ascending bellows ventilator had been used. The Tafonius piston moves to maintain zero airway pressure during expiration at a rate that is driven by recoil of the patient’s chest. Negative pressure is never generated, but potentially results in less resistance than that generated by the weight of an ascending bellows. The effect of this has not been investigated in horses to the authors’ knowledge.

In conclusion, the results of this study demonstrate that horses ventilated with FLEX require less PEEP to maintain improvements in respiratory system compliance and arterial oxygenation obtained following alveolar recruitment when compared to conventional VCV. This resulted in significantly better cardiovascular conditions in these horses. Further studies will help determine if similar results can be obtained in clinical cases.

## Data availability statement

The original contributions presented in the study are included in the article/supplementary material, further inquiries can be directed to the corresponding author.

## Ethics statement

The animal study was reviewed and approved by Institutional Animal Care and Use Committee of the University of Pennsylvania (protocol no. 806775-aaecgbc).

## Author contributions

JB contributed to the grant proposal, data collection, and manuscript preparation. KH was responsible for project conception and design, data collection, and statistical analysis. MM contributed to the grant proposal, data collection, and manuscript revision. HD was involved in data analysis and manuscript preparation. All authors contributed to the manuscript revision, read, and approved the submitted version.

## Funding

Funding for this project was provided by Raker-Tulleners Funds, New Bolton Center Raymond Firestone Trust, University of Pennsylvania.

## Conflict of interest

The authors declare that the research was conducted in the absence of any commercial or financial relationships that could be construed as a potential conflict of interest.

## Publisher’s note

All claims expressed in this article are solely those of the authors and do not necessarily represent those of their affiliated organizations, or those of the publisher, the editors and the reviewers. Any product that may be evaluated in this article, or claim that may be made by its manufacturer, is not guaranteed or endorsed by the publisher.

## Footnotes

^1^ https://stattrek.com/statistics/random-number-generator#table
